# Rotavirus gastroenteritis complicating meningitis caused by *Bacteroides uniformis* detected using mNGS: a case report and literature review

**DOI:** 10.3389/fmed.2025.1601953

**Published:** 2025-09-09

**Authors:** Ze-zheng Cai, Dong-mei Zeng, Lu-wen Lei, Sa Xiao

**Affiliations:** ^1^People’s Hospital of Qiandongnan Miao and Dong Autonomous Prefecture, Kaili, China; ^2^The Sixth Affiliated Hospital, School of Medicine, South China University of Technology, Foshan, China

**Keywords:** rotavirus gastroenteritis, meningitis, *Bacteroides uniformis*, MNGs, case report, literature review

## Abstract

This case report describes a rare instance of pediatric meningitis caused by *Bacteroides uniformis (B. uniformis)* following rotavirus gastroenteritis in a 1-year-4-month-old boy, diagnosed using metagenomics next-generation sequencing (mNGS). *Bacterial* meningitis (BM) is a life-threatening disease in children, particularly those under 5 years old, and early identification of the pathogen is crucial for reducing mortality and improving prognosis. *B. uniformis*, a Gram-negative, non-spore-forming, obligate anaerobic bacillus and common gut commensal, is rarely implicated in human infections, particularly pediatric meningitis. The child presented with vomiting, diarrhea, convulsions, and syncope, and was initially treated for meningitis and rotavirus gastroenteritis. Despite negative bacterial cultures, mNGS identified *B. uniformis* in the cerebrospinal fluid (CSF). Treatment was switched from ceftriaxone to meropenem (0.45 g, IV every 8 h) based on its good blood–brain barrier penetration and likely susceptibility of *B. uniformis*. The child’s condition improved significantly, with follow-up lumbar puncture showing normal CSF parameters and no detectable pathogens. The case suggests that rare anaerobic meningitis may occur against the backdrop of rotavirus gastroenteritis and underscores the importance of using mNGS for accurate pathogen detection in bacterial meningitis, as well as the need for early initiation of appropriate antimicrobial therapy.

## Introduction

Bacterial meningitis (BM) is a life-threatening infectious disease in children, especially those under 5 years old. In China, a nationwide retrospective study showed that from 2016 to 2021, children hospitalized for bacterial meningitis accounted for 0.22% (16,566/7,647,598) of all hospitalized cases, among whom children aged 1–5 years accounted for 35.85% (1). Rotavirus gastroenteritis, caused by the leading viral pathogen of severe diarrheal illness in young children worldwide, typically follows a benign course with intestinal symptoms predominantly (2). However, emerging evidence suggests that viral gastroenteritis can disrupt intestinal barrier function, potentially leading to bacterial translocation from the gut microbiota (3). *Bacteroides uniformis* (*B. uniformis*), a Gram-negative, non-spore-forming, obligate anaerobic bacillus and a common commensal bacterium in the human gut and vagina. It is an opportunistic pathogen that can cause exogenous and endogenous infections (4). Unlike other *Bacteroides* species, *B. uniformis* is rarely associated with human infections, and pediatric meningitis caused by this pathogen is exceedingly rare. This raises a critical clinical question: How can a normally harmless gut commensal bacterium overcome multiple protective barriers to become a life-threatening meningeal pathogen? Traditional diagnostic methods often fail to identify such rare pathogens, particularly fastidious anaerobes like *B. uniformis*. The emergence of metagenomic next-generation sequencing (mNGS) offers unprecedented opportunities to detect unusual pathogens in complex clinical scenarios. Here, we present a rare case of pediatric meningitis caused by *B. uniformis* following rotavirus gastroenteritis, demonstrating how gut-brain translocation may occur and highlighting the crucial role of advanced diagnostic technologies in solving such diagnostic dilemmas.

## Case presentation

A 1-year-4-months-old boy was admitted to the hospital on January 12, 2025, due to vomiting and diarrhea for 2 days, associated with convulsions and one episode of syncope. The patient developed symptoms after exposure to his brother, who was experiencing vomiting and diarrhea. The illness course lasted 2 days, with the main manifestations being vomiting and diarrhea. The patient vomited 10 times a day and passed yellow, watery stools 5–6 times a day. Before admission, the patient experienced a seizure following vomiting, characterized by cyanosis of the lips, fixed gaze, and unresponsiveness, lasting about 10 s. After the seizure, the patient became drowsy. Upon admission, the patient had another seizure with similar manifestations, but the duration prolonged to about 1–2 min. The seizure was alleviated after intravenous administration of midazolam, after which the patient remained in a drowsy state. Physical examination was as follows: skin and mucous, normal; heart, lung, and abdomen, normal; neck stiffness, negative; Kernig’s sign, negative. But, based on the child’s clinical presentation, the initial diagnosis was acute gastroenteritis and meningitis (etiology pending). Subsequently, a lumbar puncture was performed, and the cerebrospinal fluid (CSF) and blood was sent for biochemical analysis, routine examination, microbiological culture, and second-generation metagenomic sequencing. Empirical treatment was then initiated with ceftriaxone (0.55 g IV every 8 h), mannitol, *Bacillus subtilis* and *Enterococcus faecalis* granules, smectite powder, oral rehydration salts (type III), and intravenous fluid therapy.

Subsequently, imaging, and laboratory testing after admission were as follows: head and chest CT, No abnormalities found; CSF pressure, 80 mmH_2_O; CSF color, milky white; Pandy’s test, positive; CSF white blood cell (WBC) count, 1 × 10^6^/L; protein, 0.36 g/L; CSF glucose, 3.56 mmol/L; random blood glucose, 4.6 mmol/L; blood WBC count, 9.22 × 10^9^/L; neutrophil percentage, 53.4%; neutrophil count, 4.93 × 10^9^/L; C reactive protein (CRP): 1.0 mg/L; procalcitonin, 0.757 ng/mL; electrolytes Na^+^ 132.11 mmol/L, Cl^−^ 94.7 mmol/L; Human rotavirus (colloidal gold method) antigen, positive; liver and kidney function, normal.

**Figure 1:**
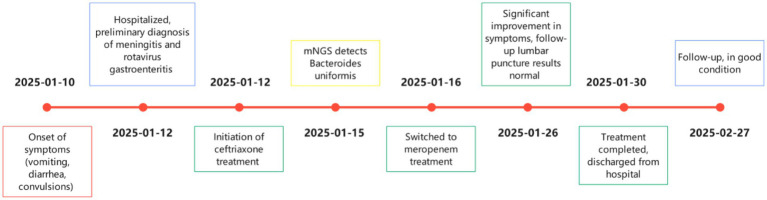
Graphical timeline.

On January 13, the child vomited 4 times, had one convulsion, and had diarrhea 8 times. On January 14, the child vomited once and had diarrhea 4 times, but the electroencephalogram (EEG) examination showed no significant abnormalities. During this period, the child’s mental state and appetite were poor, and the child was somnolent. On January 15, the CSF bacterial culture was negative, but CSF mNGS (WY309199234, Hangzhou Euroimmun Ivy Medical Laboratory Co., Ltd., Hangzhou, China) results showed that *B. uniformis* was detected (Sequence count 10,810, Relative abundance 2.35%, Coverage 6.6381%, Mean depth 1.2704×), and the fungi and *mycobacterium tuberculosis* were not detected. Based on the child’s clinical response to treatment and CSF mNGS report, the child was diagnosed with meningitis caused by *B. uniformis*. In the absence of drug sensitivity testing, the clinical pharmacist deemed that *B. uniformis* might be susceptible to meropenem. Therefore, the child was switched to meropenem (0.45 g intravenous infusion every 8 h) starting from January 16. On the second day of meropenem treatment, the child’s mental state improved, appetite returned, and there was no vomiting or diarrhea. On the 11th day of meropenem treatment, a follow-up lumbar puncture was performed. The CSF was colorless and transparent, with a negative Pandy’s test, a white blood cell (WBC) count of 0.001 × 10^9^/L, and all other results were normal. The CSF was also sent for mNGS, but no bacteria, fungi, or *mycobacterium tuberculosis* were detected. After completing 14 days of meropenem treatment, the child was discharged from the hospital when stable and in good condition. During the one-month follow-up, the child remained in good condition (The diagnostic and therapeutic process is shown in Figure 1).

## Discussion and conclusion

Meningitis is a potentially life-threatening condition characterized by infection or inflammation of the central nervous system. It is classified as bacterial, viral, or aseptic. Delayed or untreated bacterial meningitis is associated with high morbidity and mortality (5).

The clinical features of pediatric bacterial meningitis include symptoms of infection, increased intracranial pressure, and meningeal irritation, such as lethargy, abnormal crying, high-pitched crying, convulsions, poor feeding, meningeal irritation signs of the fontanelle, with or without fever, and cerebrospinal fluid leak (6). When meningitis is clinically suspected, a lumbar puncture should be performed, and a head CT scan should be conducted prior to that (7). CSF should be assayed for cell count, protein, glucose, culture, Gram stain, etc. (6, 8)The CSF of children with meningitis is usually turbid or purulent. The CSF white blood cell count is generally >100 × 10^6^/L, with neutrophils being the predominant cell type. The CSF protein level is elevated (usually >1,000 mg/L), and the CSF glucose level is decreased (usually <2.2 mmol/L) (6). In this case, the child’s CSF white blood cell count, protein level, and glucose level were all normal. Both CSF and blood cultures were negative. Brain CT scan and EEG showed no abnormalities. However, the child’s clinical presentation, including recurrent seizures, altered consciousness, and feeding difficulties, are recognized as important clinical features suggestive of bacterial meningitis in children. While viral meningitis can present with similar symptoms, the severity of neurological involvement and the rapid progression observed in this case are more characteristic of bacterial etiology.

Rotavirus can cause various neurological manifestations, including mild encephalopathy with reversible splenial lesions (MERS), acute encephalopathy, and seizures in pediatric cases (9). While rotavirus-associated CNS complications typically result from indirect mechanisms such as inflammatory mediators or metabolic disturbances, our case suggests a different pathophysiological pathway involving secondary bacterial infection (10). However, the detection of high-abundance *B. uniformis* sequences in the CSF, negative viral in the CSF, and the specific response to antianaerobic therapy support a bacterial etiology rather than direct viral CNS involvement. Therefore, the child was diagnosed with *B. uniformis* meningitis.

There have been two cases of bacterial meningitis following RV infection have been previously reported in immunocompetent children, with the causative agents being *Listeria monocytogenes* (11) and *Escherichia coli* (12), respectively. The pathophysiological mechanism underlying this cases likely involves rotavirus-induced intestinal barrier dysfunction leading to bacterial translocation. Rotavirus infection primarily targets mature enterocytes, causing villus atrophy, enhanced epithelial cell turnover, and disruption of tight junction integrity (13). Studies have demonstrated that rotavirus infection alters paracellular permeability and reduces cellular ATP levels, conditions known to compromise epithelial barrier function (14). This disruption creates a permissive environment for bacterial translocation, allowing gut commensals to cross the intestinal barrier and enter systemic circulation (3). The progression from “gut to brain” in our patient likely followed this sequence: (1) Rotavirus infection damaged intestinal epithelial barrier function and altered gut microbiota composition (2). (2) Compromised barrier function facilitated *B. uniformis* translocation into the portal circulation (3). (3) Hematogenous spread allowed the organism to breach the blood–brain barrier; (4) Local CNS infection triggered the observed neurological symptoms. This mechanism is supported by studies showing that malnutrition and enteric infections can exacerbate bacterial translocation and systemic complications (15). Through this case, we have deepened our understanding of ectopic infections by gut commensals and demonstrated the pivotal role of mNGS in resolving such diagnostic dilemmas.

*B. uniformis* is part of the *Bacteroides* genus, a significant component of the human gut microbiota, contributing to various beneficial functions (16). It is one of the least commonly isolated anaerobic bacterial species from clinical specimens (17). It is one of the least commonly isolated anaerobic bacterial species from clinical specimens. *B. uniformis* is generally considered beneficial but can spread to other parts of the body, causing infections (4). Case reports show *B. uniformis* causes diverse infections, shown in Table 1.

**Table 1:** Basic characteristics of infection caused by *Bacteroides uniformis*.

Gender	Ages (years)	Test method	Symptom	Complication	Antibiotic treatment	Outcome	Reference
Male	76	Blood cultures	Recurrent fever and acute renal failure	Portal vein thrombosis	Meropenem 1 g tid × 3 weeks	Recovered	(18)
Male	78	Blood cultures	Fatigue, anorexia, malaise, fever, chills, and low back pain without a radicular component	Spontaneous spondylodiscitis	Imipenem + metronidazole × 4 weeks, then amoxicillin–clavulanic acid×2 weeks	Recovered	(19)
Male	56	Pustule contents culture	Draining ulcer, swelling and warmth of the entire foot, fever	Skin and soft tissue infection, osteomyelitis	Cefoxitin × 18 days	Recovered	(20)
Male	1	CSF mNGS	Vomiting, convulsions, and seizures	Meningitis	Meropenem 0.45 g tid × 2 weeks	Recovered	The case

A 76-year-old male patient, who presented with recurrent fever and acute renal failure, had positive blood cultures for *Bacteroides uniformis, Escherichia coli*, and *Leptospira* spp. (18) Another case involved *B. uniformis*—induced spontaneous spondylodiscitis, where the patient had spinal infection symptoms and blood cultures positive for *B. uniformis* (19). There is also a case of *B. uniformis* and two other bacteria causing skin, soft tissue infection, and osteomyelitis (20).

Anaerobes are common pathogens in human infections. Their demanding requirements for culture conditions result in low positive detection rates. Moreover, the lengthy time required for species isolation and identification, as well as their frequent coexistence with other pathogens, pose numerous limitations for routine anaerobic culture in clinical microbiology laboratories (21). The emergence of mNGS theoretically enables comprehensive and unbiased detection of all pathogens present in clinical specimens, especially rare, emerging, or atypical pathogens in complex infectious diseases. It offers the advantages of high sensitivity, accuracy, and short detection time (22). However, this method also has some limitations, with the main drawback being the inability to perform antimicrobial susceptibility testing on detected pathogens. An survey (2005–2007) found *B. uniformis* had low resistance to most β-lactam drugs, and no resistance to piperacillin-tazobactam and linezolid (23). A tertiary hospital’s retrospective study (2010–2022) on 12 non-fragilis *Bacteroides* strains showed 50% sensitivity to amoxicillin-clavulanate, 100% to imipenem-cilastatin (24). An Irish university hospital’s study (2010–2020) on 688 *Bacteroides* spp. strains revealed an overall resistance rate of 32.79%, but 0 to meropenem (25).

The management of meningitis includes the early initiation of antimicrobial agents that can penetrate the blood–brain barrier. If possible, antimicrobial therapy should be initiated within 1 h after the patient arrives at the hospital, and it is recommended to avoid delaying antimicrobial administration beyond 3 h after presentation (6). Ceftriaxone or cefotaxime in combination with vancomycin is recommended (6, 26). Other antimicrobial agents that can achieve good concentrations in the CSF include high-dose penicillin G, meropenem and so on (6). Given meropenem’s good penetration of the blood–brain barrier and high sensitivity, ceftriaxone was discontinued and replaced with meropenem. After 14 days of anti-anaerobic treatment with meropenem, combined with mannitol to reduce intracranial pressure, the child’s symptoms were significantly alleviated.

The uniqueness of this case lies in *B. uniformis* meningitis occurring secondary to rotavirus gastroenteritis, a relatively rare secondary infection possibly related to intestinal barrier dysfunction. The case suggests that rare anaerobic meningitis may occur against the backdrop of rotavirus gastroenteritis, but more research is needed to confirm this association. Therefore, more attention should be paid to anaerobe detection in bacterial meningitis, and mNGS can accurately detect infectious pathogens.

## Data Availability

The datasets presented in this study can be found in online repositories. The names of the repository/repositories and accession number(s) can be found in the article/supplementary material.
