# Correction: Rotavirus gastroenteritis complicating meningitis caused by *Bacteroides uniformis* detected using mNGS: a case report and literature review

**DOI:** 10.3389/fmed.2025.1703987

**Published:** 2025-09-22

**Authors:** Frontiers Production Office

**Affiliations:** Frontiers Media SA, Lausanne, Switzerland

**Keywords:** rotavirus gastroenteritis, meningitis, *Bacteroides uniformis*, mNGS, case report, literature review

Author Ze-zheng Cai was erroneously assigned as corresponding author. The correct corresponding authors are listed below:

^*^Correspondence:

Sa Xiao

xiaosa_sophia@163.com

Lu-wen Lei

leiluwen618@163.com

The original version of this article has been updated.

